# 24-Month Registry Results on Drug-Coated Balloon Angioplasty Below the Knee: Strong Enough Evidence to Convince the Sceptics?

**DOI:** 10.1007/s00270-020-02620-4

**Published:** 2020-09-23

**Authors:** Ulf Teichgräber

**Affiliations:** Department of Radiology, Jena University Hospital, Am Klinikum 1, 07747 Jena, Germany

Critical limb ischemia (CLI) requires immediate revascularization to prevent limb loss. In less-complex disease and in patients with severe comorbidities, the endovascular approach can be the preferred strategy. However, so far, research on Paclitaxel drug-coated balloon (DCB) angioplasty of below-the-knee (BTK) arteries gives rise to controversy.

Initially, the single-center DEBATE-BTK study (65 DCB vs. 67 POBA participants) suggested a potential advantage of DCB angioplasty over standard balloon angioplasty (plain old balloon angioplasty; POBA) [1]. DCB significantly reduced the incidence of 12-month binary restenosis and target lesion revascularization (TLR). No major amputation was needed. In contrast, the larger, multi-center IN.PACT DEEP trial that evaluated the same DCB (IN.PACT Amphirion) failed to meet superiority of DCB over POBA and showed only comparable 5-year effectiveness (follow-up completed in 95 DCB and 49 POBA participants). The tendency to an increased rate of major amputations with DCB angioplasty at 12 months weakened throughout 5-years (15.4% DCB vs. 10.6% POBA; *p* = 0.11) [2]. The randomized BIOLUX P-II trial (36 DCB vs. 36 POBA participants) presented 12-month results on the Passeo-18 BIOLUX DCB and also confirmed only comparable effectiveness between DCB and POBA without any safety signal from DCB (major amputation: DCB 3.3% vs. POBA 5.6%; *p* = 0.63) [3]. Finally, 12-month outcomes from the APOLLO observational study on the ELUTAX SV DCB in CLI patients (164 participants) revealed estimates of primary patency and freedom from TLR of 68.5% and 90.6%, respectively. Incidence of major amputation and mortality was 5% and 15.9%, respectively [4].

In this issue of CVIR, Tepe et al. present 24-month results of DCB angioplasty in a pre-specified subgroup of 151 participants with BTK lesions from the real-world BIOLOUX P-III registry [5]. Most of the participants had CLI (76.0%) with a share of 22.3% categorized as Rutherford-Becker class 6. A total of 62.9% participants were diabetics. Mean lesion length was 7.9 cm, proportionate share of total occlusions 18.4%, and that of severely calcified lesions 9.8%. Lesion preparation was conducted in 73.0% and bailout stenting in 1.1% of lesions. No data are provided whether inflow vessels were diseased or treated. At 24 months, primary patency and freedom from TLR were favorable (82.8% and 90.9%, respectively), however, against the background of 9.9% major amputations and 20.8% mortality.

Overall, Tepe et al. provided valuable data on effectiveness of DCB, however, no direct comparison with POBA, the standard of care, which would be needed to prove efficacy. The range of 12-month effectiveness across DCB studies for BTK lesions is large (primary patency 49.2–86.6%; freedom from TLR 68.7–90.6% [3, 5]). This also applies to safety (major amputation 0.0–9.0%; mortality 8.8–15.9% [1, 2, 4]). With regard to safety, neither the recent study of Tepe et al. nor any of the earlier studies were powered to compare major amputation or all-cause mortality rates that should be seen even in light of possible paclitaxel embolization and its potential adverse side effects.

Whether differences are due to patient risk, limb threat according to wound grading, anatomic complexity, lesion preparation, or to index procedure and DCB type can only be imagined. However, except for mortality, the recent 24-month results suggest a relatively stable course of the disease throughout the second year after index procedure. After all, 5-year mortality in the IN.PACT DEEP trial was similar across groups (DCB 39.4% vs. POBA 44.9%) and mortality might comply with the advanced stage of the disease.

In summary, both efficacy and safety of DCB in BTK lesions are still to be evaluated with appropriately controlled, adequately powered trials and long-term follow-ups. Until then, POBA should remain standard of care when infra-popliteal endovascular revascularization is indicated.
